# Tumor-Agnostic Landscape with *HER2* Amplification in Japan: Real-World Prevalence and Implications for Targeting *HER2*

**DOI:** 10.3390/curroncol33040195

**Published:** 2026-03-30

**Authors:** Yutaka Hatanaka, Jun Sasano, Osamu Takizawa

**Affiliations:** 1 Center for Development of Advanced Diagnostics, Hokkaido University Hospital, Sapporo, Hokkaido 060-8648, Japan; 2 Oncology Medical Science Department, Daiichi Sankyo Co., Ltd., Chuo-ku, Tokyo 103-8426, Japan; 3 Data Intelligence Department, Daiichi Sankyo Co., Ltd., Shinagawa-ku, Tokyo 140-0005, Japan

**Keywords:** comprehensive genomic profiling, human epidermal growth factor receptor 2 amplification, next-generation sequencing, prevalence, solid tumors

## Abstract

Limited data exist on the prevalence of human epidermal growth factor receptor 2 (*ERBB2*/*HER2*) amplification in patients with all types of solid tumors. This retrospective, observational study (UMIN ID: UMIN000057382) analyzed the prevalence of *HER2* amplification across all solid tumors using comprehensive genomic profiling (CGP) data from the Center for Cancer Genomics and Advanced Therapeutics database in Japan. We analyzed 89,374 eligible patients with solid tumors: *HER2* amplification was detected in 5119 patients (5.7%). The highest rates of *HER2* amplification were observed in patients with tumors of the esophagus/stomach (12.9%), followed by tumors of the bladder/urinary tract (10.6%), breast (9.5%), biliary tract (8.4%), and uterus (8.4%). Among the five assay platforms, FoundationOne CDx accounted for 69.7% of all CGP tests and had an *HER2* amplification detection rate of 7.4%, compared to the other four platforms (range: 1.9–15.4% of all CGP tests [detection rates: 1.5–2.3%]). Substantial differences were observed in the mutation frequencies of multiple genes between *HER2* amplified and *HER2* non-amplified tumors. The results highlight that *HER2* amplification extends beyond conventional tumor types and enables the identification, via CGP testing, of non-traditional tumor subsets (cancers other than breast and gastric cancer), including rare cancers, that could be candidates for *HER2*-targeting therapy such as trastuzumab deruxtecan in Japan.

## Simple Summary

Considering the availability of human epidermal growth factor receptor 2 (*ERBB2/HER2*)-targeting therapy, the status of *HER2* amplification in patients with all types of solid tumors needs to be clarified. We analyzed 89,374 eligible patients with solid tumors using comprehensive genomic profiling data from Japanese nationwide database, and *HER2* amplification was detected in 5119 patients (5.7%): highest rates of *HER2* amplification were observed in patients with tumors of the esophagus/stomach (12.9%), followed by tumors of the bladder/urinary tract (10.6%), breast (9.5%), biliary tract (8.4%), and uterus (8.4%). The detection of *HER2* amplification was highest with FoundationOne CDx assay platform (7.4%) compared to other platforms. We report that *HER2* amplification occurs in several tumors, including rare tumors, and is not limited to conventional tumor types. The results suggest that there is a need to expand the evaluation of *HER2* amplification and consequently *HER2*-targeting therapy such as trastuzumab deruxtecan in Japan.

## 1. Introduction

Human epidermal growth factor receptor 2 (*HER2*, also known as *ERBB2*) is a proto-oncogene that encodes a transmembrane tyrosine kinase receptor involved in the regulation of cell growth, differentiation, and survival [1]. *HER2*-positive tumors are identified by either protein overexpression (using immunohistochemistry with a score of 3+ [IHC 3+], which is the current standard) or IHC 2+ with concurrent gene amplification, confirmed by in situ hybridization (ISH) [2]. Thus, both amplification and overexpression of *HER2* are established therapeutic targets, particularly in breast and gastric cancers [1,3].

In breast cancer, *HER2* amplification occurs in approximately 20% of cases, while 15–20% of cases show *HER2* overexpression, which is linked to aggressive tumor characteristics [3,4]. However, this subset now has effective treatment options due to *HER2*-targeted therapies (such as trastuzumab, pertuzumab) and newer drugs (such as trastuzumab deruxtecan [T-DXd]) [1]. Similarly, *HER2* amplification is observed in 11–16% of gastric/gastroesophageal cancers, while *HER2* overexpression in about 20% of cases, and anti-*HER2* therapies have demonstrated clinical benefits [1,4].

In addition to these tumor types, *HER2* amplification has also been increasingly recognized in various other solid tumors, including colorectal cancer, non-small cell lung cancer (NSCLC), biliary tract cancer, and bladder cancer, although at lower frequencies [4,5]. However, the overall landscape of *HER2* amplification across the full spectrum of solid malignancies remains unclear.

For the treatment of patients with advanced *HER2*-amplified solid tumors, the HERALD trial conducted in Japan demonstrated the efficacy of T-DXd [6]. However, the prevalence of *HER2* amplification detected by comprehensive genomic profiling (CGP) testing in Japan, as well as differences among assay platforms, have not yet been clearly defined. Understanding these aspects will become increasingly important in clinical practice for precision oncology.

CGP, based on next-generation sequencing (NGS), offers a broader approach for detecting multiple genomic alterations, including *HER2* amplification, across tumor types and patient populations. In Japan, the Center for Cancer Genomics and Advanced Therapeutics (C-CAT) serves as a centralized database that integrates genomic and clinical information from patients who have undergone CGP under the National Cancer Genomic Medicine Program [7,8].

Large-scale genomic databases such as AACR Project GENIE have reported *HER2* amplification frequencies of about 12% for esophagogastric cancer, 10% for breast cancer, and <4% for all other cancers. However, such prevalence estimates are derived largely from Western populations and may not reflect the genomic landscape in Asian patients, particularly in Japan [9,10]. Additionally, estimates can vary depending on the differences in the assay platforms used.

Overall, there is a need for genomically matched treatment with awareness of the prevalence of *HER2* amplification in patients with all types of solid tumors without standard treatment or in patients with locally advanced or metastatic tumors who have completed standard treatment (including those who are expected to complete treatment). In Japan, there is a lack of Japan-specific real-world data regarding *HER2* amplification using CGP testing.

Therefore, the primary objective of this study was to determine the prevalence of *HER2* amplification across all solid tumors using CGP data from the C-CAT database in Japan. In addition, background information, including treatment histories and genetic mutations, was compared between patients with and without *HER2* amplification.

## 2. Materials and Methods

### 2.1. Study Design and Data Source

This retrospective observational study (UMIN ID: UMIN000057382) evaluated the CGP data of patients with solid tumors from the C-CAT database, Japan’s national repository integrating genomic and clinical data under the National Cancer Genomic Medicine Program. Patient data were extracted and analyzed based on CGP test results recorded between 1 June 2019 and 31 December 2024 (database lock: May 2025). This large database (111,560 patients registered at the end of August 2025) includes 289 medical institutions certified by the Ministry of Health, Labour and Welfare as core cancer genome medical centers, core hospitals, and affiliated hospitals (as of 1 October 2025).

The observation period initiation date (index date) was based on the “expert panel (EP) held” date in the C-CAT database. Since the response to treatment at each facility is likely to differ based on the results of each CGP investigation, the information before the index date was regarded as background information, and the information after the index date was regarded as information from the follow-up period.

Informed consent for secondary data use by other parties was obtained from each patient prior to data transfer from their respective hospitals. Of note, >99% of patients in the C-CAT database have agreed to the secondary use of their genomic and clinical information, and all data extracted for this study were from patients who provided informed consent for secondary data use [11,12]. The CGP database included individual patient data and information on gene alterations, drug therapy (before and after EP), CGP specimens, and outcomes. CGP data were obtained using five platforms: tissue-based CGP (tissue CGP using tissue DNA) included the Oncoguide™ NCC OncoPanel System (NOP), FoundationOne^®®^ CDx (F1CDx), and GenMineTOP^®®^ (GMTOP), whereas plasma-based CGP using liquid biopsy (plasma CGP using circulating tumor DNA/ctDNA) included FoundationOne^®®^ Liquid CDx (F1LCDx) and Guardant360^®®^ CDx (G360CDx).

The study was approved by the Ethical Research Practice Committee for Research on Human Tissue, Information, and Other Human Material (Approval No. 000790) and reviewed by the C-CAT Data Utilization Review Board (Approval ID: CDU2024-026N) [13].

Individual patient data in this study were provided to the C-CAT database vendors as anonymously processed information by individual medical institutions and did not include personally identifiable information. Of note, in the C-CAT database, a genomic sequence falls under the personal identification code, and it is classified into the “personal information requiring consideration” category because it contains disease information. Patient consent, including consent for secondary data use prior to CGP investigation, was obtained from all facilities.

### 2.2. Patients

Eligible patients were required to be ≥18 years of age, enrolled in the C-CAT database during the study period (1 June 2019 to 31 December 2024), and having solid tumors. Patients were excluded if they had a record of a previous registration to avoid duplication of data, an unknown previous registration status, and CGP results including *HER2* amplification that could not be judged or were indeterminate, i.e., not all alterations and co-alterations were detected, and the presence or absence of *HER2* amplification was unknown. Eligible patients, with or without *ERBB2* amplification, enrolled in the C-CAT database during the study period were categorized into the *HER2* amplification and *HER2* no-amplification groups, respectively. In this study, *HER2* amplification was defined as the presence or absence of records in the C-CAT database and was not based on the copy number, because the copy number was not recorded in this database. Additionally, other *HER2* alterations (mutations [such as single-nucleotide variants] and fusions) apart from amplification might have been included in both groups. Tumor types were classified according to OncoTree, which includes different histological and genomic subclassifications [14].

### 2.3. Endpoints

The primary endpoint was the prevalence of *HER2* amplification using CGP test results. The secondary endpoints were the detection rates of *HER2* amplification by CGP assay modality, patient background characteristics based on *HER2* amplification (e.g., age, sex, tumor type, metastasis site, number of treatment lines before EP, and CGP platform type), and co-occurring gene alterations using CGP. The exploratory endpoint was to assess the implication of CGP results on the direction of treatment after EP, i.e., application of genomically matched treatment.

### 2.4. Statistical Analyses

Descriptive statistics were used to summarize categorical and continuous data, which are presented as mean and standard deviation (SD), and categorical data are presented as frequency and percentage. Chi-square tests were performed as necessary. All analyses were performed in accordance with the Strengthening the Reporting of Observational Studies in Epidemiology guidelines [15] and relevant sections of the Consolidated Standards of Reporting Trials guidelines [16]. Tumor types were categorized according to the OncoTree hierarchical classification system into the primary level (Level 1), secondary level (Level 2), tertiary level (Level 3), and quaternary level (Level 4), based on histological and genomic subclassifications. This allows meaningful comparisons of prevalence data across tumor subtypes for real-world interpretation and subsequent targeted therapies [14].

Patient characteristics were also evaluated, and patients were categorized into the *HER2* amplification and *HER2* no-amplification groups for all tumor types, with data presented using descriptive statistics. The percentages of tumor types with genetic mutations, *HER2* amplification, and the frequency of co-occurring gene mutations [8] were visualized using a heat map. Missing data imputation was not performed. All analyses were performed using the SAS software package (version 9.4; SAS Institute Inc., Cary, NC, USA) and Python (version 3.11, Python Software Foundation, Wilmington, DE, USA).

## 3. Results

### 3.1. Patient Disposition and Baseline Characteristics

**Figure 1:**
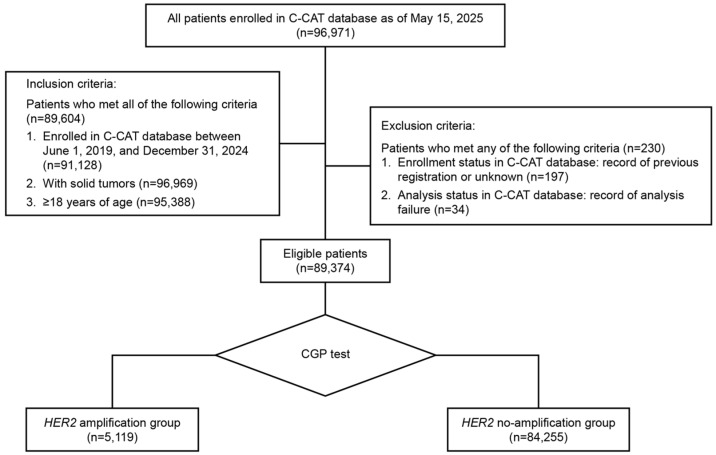
Patient flow diagram. C-CAT: Center for Cancer Genomics and Advanced Therapeutics; CGP: comprehensive genomic profiling; *HER2*: human epidermal growth factor receptor 2.

As of 15 May 2025 (C-CAT database ver.20250416), the records of 96,971 patients enrolled in the C-CAT database were evaluated. The analysis set comprised 89,374 eligible patients with solid tumors categorized into the *HER2* amplification (*n* = 5119) and *HER2* no-amplification (*n* = 84,255) groups, using evaluable CGP results (Figure 1).

The commonest tumors (Level 1) identified in the database were tumors of the bowel (16.7% [14,888/89,374]), followed by those of the pancreas (15.6% [13,976/89,374]), biliary tract (8.9% [7966/89,374]), and breast (7.5% [6720/89,374]) (Table S1). Most of the patients presented with metastasis (89.5% [79,962/89,374]) before the index date (*HER2* no-amplification group [89.2%] vs. *HER2* amplification group [93.6%]), and the commonest site of metastasis was lymph nodes/lymph vessels (35.6% [31,774/89,374]), followed by the liver (32.9% [29,410/89,374]), lung (28.9% [25,792/89,374]), peritoneum (19.7% [17,595/89,374]), and bone (17.7% [15,838/89,374]) (Table 1). Most samples used in tissue CGP were collected surgically (61.0% [45,137/73,960]), followed by biopsies (38.6% [28,566/73,960]). A biopsy was performed at a higher rate in patients with prostate cancer (78.6% [2627/3341]), followed by those with other tumors (62.0% [1223/1971]) and lung cancer (61.7% [2408/3903]; Table S1). Biopsy specimens were collected from the primary lesion site in 68.2% (50,458/73,960) of patients and metastatic lesions in 31.3% (23,169/73,960) of patients (Table 1). The tumor type with the highest rate of plasma CGP was prostate cancer (39.4% [2169/5510]), followed by pancreatic cancer (28.9% [4033/13,976]) and lung cancer (27.9% [1514/5417]). When categorized by line of treatment (1st to ≥5th lines), the proportion of patients was almost evenly distributed among all treatment lines for overall tumor types. A higher proportion of patients with rare tumors underwent CGP testing after receiving first-line and second-line treatments compared with patients having other tumors. On the other hand, CGP testing was commonly performed after third-line treatment for tumor types with several treatment options (Table S1).

### 3.2. Prevalence of HER2 Amplification

*HER2* amplification was detected in 5.7% (5119/89,374) of eligible patients with solid tumors.

**Figure 2:**
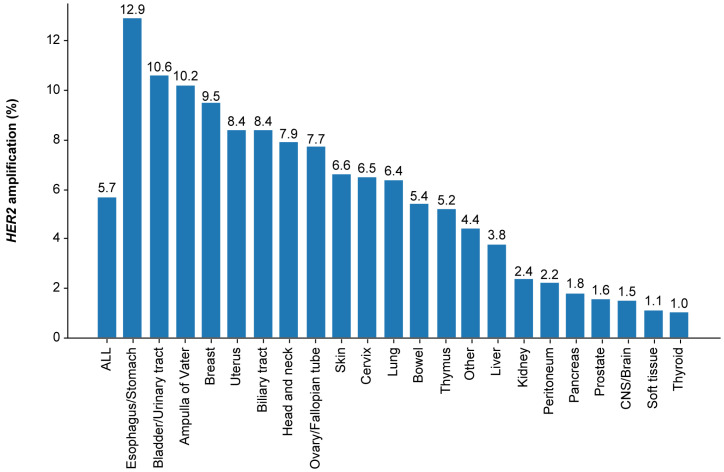
Prevalence of *HER2* amplification in solid tumors. CNS, central nervous system; *HER2*: human epidermal growth factor receptor 2.

The *HER2* amplification rate was highest in patients with tumors of the esophagus/stomach (12.9% [709/5499]), followed by those of the bladder/urinary tract (10.6% [157/1484]), ampulla of Vater (10.2% [54/531]), breast (9.5% [639/6720]), biliary tract (8.4% [673/7966]), and uterus (8.4% [258/3068]) (Figure 2 and Table S2).

### 3.3. Prevalence of HER2 Amplification by the OncoTree Hierarchical Classification System

The prevalence of *HER2* amplification in tumors of the esophagus/stomach (Level 1) was 12.9% (*n* = 709/5499), with Level 2 prevalence in esophagogastric adenocarcinoma of 16.1% (*n* = 491/3041) and esophageal squamous cell carcinoma of 6.6% (*n* = 95/1440), and Level 3 prevalence in gastroesophageal junction adenocarcinoma of 21.5% (*n* = 73/339) and stomach adenocarcinoma of 17.0% (*n* = 353/2074).

The prevalence of *HER2* amplification in tumors of the bowel (Level 1) was 5.4% (*n* = 803/14,888), with Level 2 prevalence in colorectal adenocarcinoma of 5.7% (657/11,451) and small bowel cancer of 5.3% (28/529), and Level 3 prevalence in colon adenocarcinoma of 5.6% (338/6064) and rectal adenocarcinoma of 6.1% (258/4228).

The prevalence of *HER2* amplification in tumors of the ampulla of Vater (Level 1) was 10.2% (54/531), with Level 2 prevalence in ampullary carcinoma of 10.0% (45/451).

The prevalence of *HER2* amplification in tumors of the biliary tract (Level 1) was 8.4% (673/7966), with Level 2 prevalence in intraductal papillary neoplasm of the bile duct of 6.0% (325/5455); Level 3 prevalence in gallbladder cancer of 15.6% (291/1868) and cholangiocarcinoma of 5.9% (322/5419); and Level 4 prevalence in extrahepatic cholangiocarcinoma of 9.3% (93/995), intrahepatic cholangiocarcinoma of 4.8% (136/2842), and perihilar cholangiocarcinoma of 5.4% (60/1107).

The prevalence of *HER2* amplification in tumors of the bladder/urinary tract (Level 1) was 10.6% (157/1484), with Level 2 prevalence in bladder urothelial carcinoma of 16.0% (91/568) and upper tract urothelial carcinoma of 7.9% (34/431).

The prevalence of *HER2* amplification in tumors of the cervix (Level 1) was 6.5% (141/2177), with Level 2 prevalence in cervical adenocarcinoma of 10.2% (63/617) and cervical squamous cell carcinoma of 4.7% (44/941).

The prevalence of *HER2* amplification in tumors of the uterus (Level 1) was 8.4% (258/3068), with Level 2 prevalence in endometrial carcinoma of 11.8% (201/1697) and uterine sarcoma/mesenchymal tumors of 2.3% (19/828) and Level 3 prevalence in uterine carcinosarcoma/uterine malignant mixed Müllerian tumor of 14.5% (40/276), uterine clear cell carcinoma of 18.6% (13/70), uterine endometrioid carcinoma of 6.2% (44/715), uterine serous carcinoma/uterine papillary serous carcinoma of 25.2% (70/278), and uterine smooth muscle tumor of 2.3% (12/512).

The prevalence of *HER2* amplification in tumors of the ovarian/fallopian tube (Level 1) was 7.7% (379/4941), with Level 3 prevalence in clear cell ovarian cancer of 21.4% (187/875), mucinous ovarian cancer of 13.5% (28/207), serous ovarian cancer of 4.0% (74/1856), and endometrioid ovarian cancer of 4.1% (11/270). Its Level 4 prevalence was higher in high-grade serous ovarian cancer (4.2% [61/1436]) than in low-grade serous ovarian cancer (2.4% [2/85]).

The prevalence of *HER2* amplification in tumors of the breast (Level 1) was 9.5% (639/6720), with Level 2 prevalence in invasive breast carcinoma of 9.5% (543/5704) and Level 3 prevalence in breast invasive ductal carcinoma of 9.7% (430/4422) and breast invasive lobular carcinoma of 3.1% (11/354).

The prevalence of *HER2* amplification in tumors of the lung (Level 1) was 6.4% (348/5417), with Level 2 prevalence in NSCLC of 6.8% (289/4269) and Level 3 prevalence in lung adenocarcinoma of 7.2% (228/3177), lung squamous cell carcinoma of 4.6% (24/517), large cell lung carcinoma of 14.3% (3/21), and small cell lung cancer of 2.3% (7/308).

The prevalence of *HER2* amplification in tumors of the head and neck (Level 1) was 7.9% (231/2911), with Level 2 prevalence in salivary carcinoma of 13.7% (133/972) and head and neck squamous cell carcinoma of 4.1% (37/908) and Level 3 prevalence in adenoid cystic carcinoma of 2.8% (12/425) and salivary duct carcinoma of 40.2% (86/214).

The prevalence of *HER2* amplification in tumors of the skin (Level 1) was 6.6% (91/1381), with Level 2 prevalence in melanoma of 3.3% (20/614) and extramammary Paget disease of 27.7% (41/148) (Table S2).

### 3.4. Detection Rate of HER2 Amplification by CGP Assay Modality

**Figure 3:**
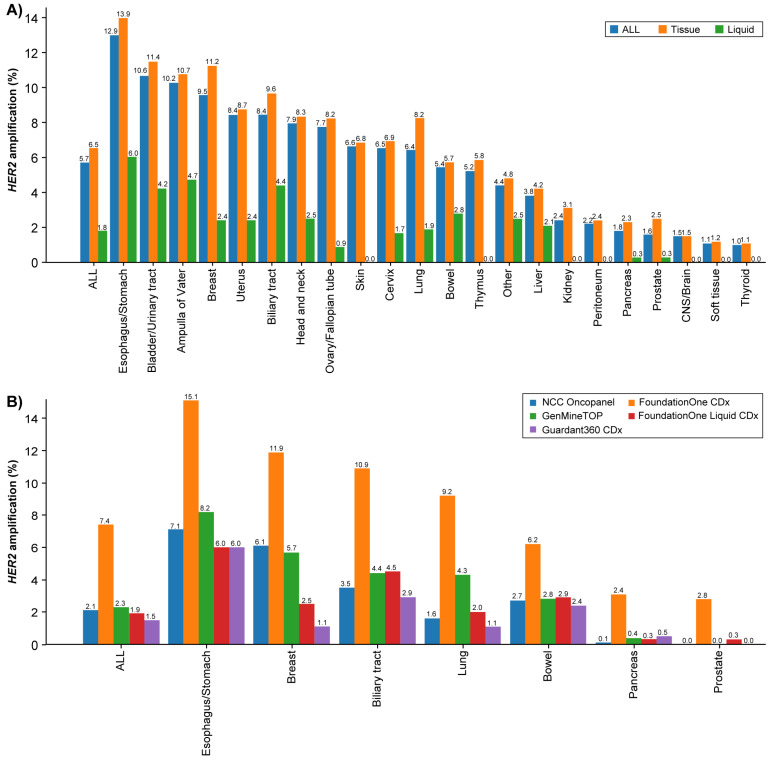
Prevalence of *HER2* amplification by CGP. (**A**) Percentage of *HER2* amplification detected according to sample type. (**B**) Percentage of *HER2* amplification by assay platforms in selected tumor types. CGP: comprehensive genomic profiling; CNS: central nervous system; *HER2*: human epidermal growth factor receptor 2.

Tissue CGP testing was predominant (82.8% [73,960/89,374]), whereas plasma CGP testing accounted for 17.2% (15,414/89,374) of all tests. *HER2* amplification was detected in 6.5% (4839/73,960) of tissue CGP, which was higher than 1.8% (280/15,414) for plasma CGP. When stratified by assay platforms, F1CDx accounted for 84.2% (62,299/73,960) of tissue CGP and F1LCDx for 89.0% (13,719/15,414) of plasma CGP. In tissue CGP, the highest rates of *HER2* amplification were observed in the esophagus/stomach (13.9% [667/4801]), bladder/urinary tract (11.4% [150/1319]), breast (11.2% [609/5461]), and ampulla of Vater (10.7% [52/488]) (Figure 3). In plasma CGP, detection rates were highest in the esophagus/stomach (6.0% [42/698]), and subsequently in the ampulla of Vater (4.7% [2/43]), biliary tract (4.4% [78/1788]), and bladder/urinary tract (4.2% [7/165]) (Figure 3A and Table S3). The *HER2* amplification detection rates by assay platform were 7.4% for F1CDx, 2.3% for GMTOP, 2.1% for NOP, 1.9% for F1LCDx, and 1.5% for G360CDx (Figure 3B and Table S3).

### 3.5. Patient Characteristics by HER2 Amplification Status

In the *HER2* amplification and *HER2* no-amplification groups, the mean [SD] age (62.5 [12.0] years [*n* = 5119]) vs. 62.5 [12.5] years [*n* = 84,255]) and proportion of smokers (44.4% [2275/5119]) vs. 44.2% [37,208/84,255]) were comparable (Table 1). Among the assay platforms, *HER2* amplification was more frequently detected with tissue CGP (particularly with F1CDx: 89.6% [4586/5119]) but was less common with plasma CGP (particularly with G360CDx: 0.5% [26/5119]) (Table 1). Patients with a record of *HER2*-positive (IHC 3+ or fluorescence in situ hybridization [FISH] or ISH) status prior to CGP testing had higher rates of *HER2* amplification, and patients who tested *HER2* negative prior to CGP testing tended to have no detectable *HER2* amplification (*HER2* no-amplification group). However, among *HER2*-positive patients, the proportion of *HER2* amplification by CGP was relatively low (IHC 3+: 61.5% [278/452] in breast, 70.8% [400/565] in esophagus/stomach; ISH-positive: 38.4% [84/219] in breast, 40.0% [16/40] in esophagus/stomach) (Table S4). Conversely, with respect to other predictive biomarkers examined prior to CGP, strong associations were observed for *KRAS* and *BRAF*V600E mutations in bowel (predominantly colorectal) cancer (*p* < 0.0001 and *p* = 0.0008, respectively), homologous recombination deficiency (HRD) in ovarian cancer (*p* = 0.0012), and programmed death-ligand 1 expression in lung cancer (*p* = 0.0074) (Table S5).

### 3.6. Mutation Frequencies of Multiple Genes from CGP Assay

**Figure 4:**
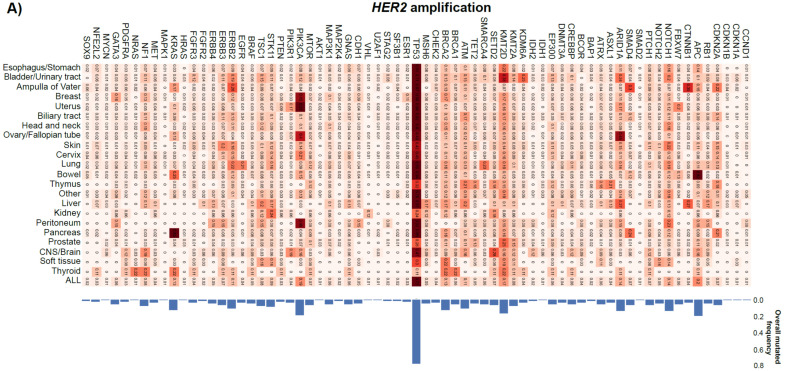

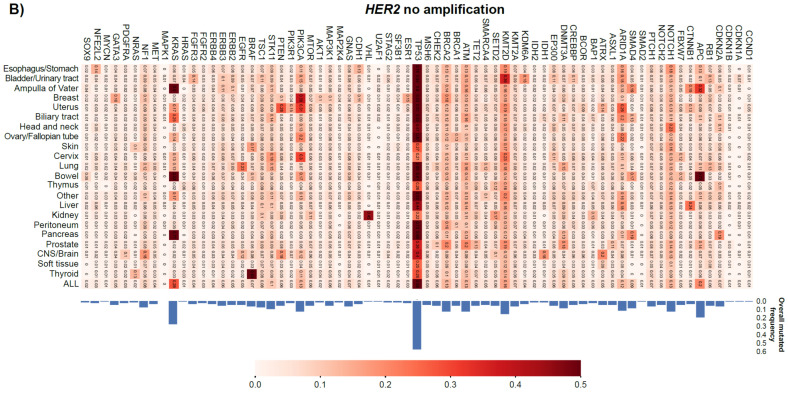
Heat map of gene mutation frequency by CGP. (**A**) *HER2* amplification group; (**B**) *HER2* no-amplification group. CGP: comprehensive genomic profiling; CNS: central nervous system; *HER2*: human epidermal growth factor receptor 2.

There were more numbers and types of co-occurring gene mutations in the *HER2* amplification group than in the *HER2* no-amplification group. *KRAS* mutations were more common in the *HER2* no-amplification group (28%) than in the *HER2* amplification group (13%). *TP53* mutations were common across tumor types in both the *HER2* amplification and *HER2* no-amplification groups. The frequency of *TP53* mutations was also similar across tumor types in both the *HER2* amplification (77%) and *HER2* no-amplification (58%) groups (Figure 4). Amplified genes in both the *HER2* amplification and *HER2* no-amplification groups were identified. In the *HER2* amplification group, co-amplification occurred in 83% of cases. The most frequent co-amplified genes were *CDK12* (33%) and *MYC* (16%). In contrast, in the *HER2* no-amplification group, the most frequent genes were *MYC* (7%), *RAD21* (5%), and *CCND1* (4%). *CDK12* gene showed the highest frequency ratio (*HER2* amplification/no-amplification group) (Figure S1). In comparison, gene fusions were infrequent (2.7%) in the *HER2* amplification group. The main driver genes were *ERBB2* (*n* = 15), *FGFR3* (*n* = 12), *FGFR2* (*n* = 6), *BRAF* (*n* = 6), *RET* (*n* = 6), *ALK* (*n* = 5), and *ERG* (*n* = 2). The fusion partners of *ERBB2* were heterogeneous, while those of other genes were largely restricted to specific partners: *TACC3* for *FGFR3*, *TACC2* for *FGFR2*, *DENND2A* for *BRAF*, *CCDC6* for *RET*, *EML4* and *HMBOX1* for *ALK*, and *TMPRSS2* for *ERG* (Table S6).

### 3.7. Status of Treatment After EP Evaluation

In the *HER2* amplification group, a genomically matched new therapeutic drug treatment was recommended by EP to 62.2% (3183/5119) of patients (recommendation range for all tumors: 38.2–69.2%). Among them, 23.5% (748/3183) were administered the recommended drug (Table 2). Furthermore, 39.3% (294/748) of them received anti-*HER2* drugs (Table S7).

In the *HER2* no-amplification group, a genomically matched new therapeutic drug treatment was recommended by EP to 38.9% (32,798/84,225) of patients (recommendation range for all tumors: 23.9–58.3%). Among them, 17.8% (5840/32,798) were administered the recommended drug (Table 2). Furthermore, 1.1% (62/5840) of them received anti-*HER2* drugs (Table S7).

## 4. Discussion

This large-scale, real-world study determined the prevalence of *HER2* amplification across a wide spectrum of solid tumors based on CGP testing using the C-CAT database. With approximately 90,000 patients included in the analysis and over 5100 *HER2* amplification cases, this study represents one of the most extensive datasets examining *HER2* amplification beyond conventionally studied tumor types such as breast and gastric cancers.

The prevalence of *HER2* amplification in all solid tumors assessed by CGP testing using NGS was 5.7%. In addition to its well-known high prevalence in esophagus/stomach (12.9%) and breast (9.5%) tumors, other major tumor types with high frequencies included tumors of the bladder/urinary tract (10.6%), biliary tract (8.4%), and uterus (8.4%). In smaller fractions with fewer than 1000 tested cases, very high amplification rates were observed in salivary duct carcinoma (a Level 3 subset of head and neck cancer; 40.2%), extramammary Paget’s disease (a Level 2 subset of skin cancer; 27.7%), and ampulla of Vater (Level 1; 10.2%). These findings confirm that *HER2* amplification is not confined to breast and gastric cancers but extends across a broader tumor spectrum including smaller fractions. There are several reports describing *HER2* amplification rates in these tumor types using NGS. Based on published NGS analyses, the reported prevalence of *HER2* amplification is 12.8% (33/256) in NSCLC [17], 11.9% (12/101) in gastric cancer, 9.4% (6/64) in esophageal cancer, 5.3% (8/151) in endometrial cancer, 5.2% (6/116) in bladder cancer, 4.9% (21/426) in biliary cancer, and 2% (26/1300) to 3.6% (30/842) in colorectal cancer [18,19]. However, due to small sample sizes, direct comparison with the present study remains difficult. Similarly, in a cohort of 37,436 sequenced cases from AACR Project GENIE, the prevalence of *HER2* amplification was approximately 12% in esophagogastric cancer, 10% in breast cancer, and <4% in all other cancers [9,10]. In another study that evaluated 429,666 solid tumors profiled using F1CDx, *HER2* amplification was found in 10.9% of patients with bladder cancer, 9.9% with breast cancer, 15.5% with gastroesophageal cancer, 3.9% with NSCLC, and 3.7% with colorectal cancer [20].

The *HER2* amplification rates observed in this study for tumors of the esophagus/stomach (12.9%) and breast (9.5%) are generally consistent with previously reported data obtained using NGS and other methods for determining amplification [1,21]. However, considering the differences in clinical practice for *HER2* testing between the United States (where testing targets overexpression by IHC) and Japan (where, for the time being, testing is expected to focus only on amplification by CGP), understanding the concordance between *HER2* gene amplification and protein overexpression is of critical clinical importance. In this study, it was possible to cross-reference CGP test results with those of IHC and ISH assays prior to CGP. Relying solely on amplification may lead to missing treatable patients, underscoring the need for cautious clinical application. This finding raises concerns based on the observation that the prevalence of *HER2* amplification by CGP among *HER2* IHC 3+ or ISH-positive cases was not necessarily high in breast and esophagus/stomach cancers. However, these comparisons have limitations, such as cases in which different specimens were used at the time of testing.

In this study, CGP testing was predominantly performed with tissue CGP (82.8%), rather than plasma CGP (17.2%), and the prevalence of *HER2* amplification was 6.5% with tissue CGP compared with 1.8% with plasma CGP. In addition, the detection rates by assay platform were 7.4% for F1CDx, 2.1% for NOP, 2.3% for GMTOP, 1.9% for F1LCDx, and 1.5% for G360CDx, indicating that the prevalence of *HER2* amplification in this C-CAT cohort was substantially influenced by the detection rate of F1CDx. Furthermore, to clarify the details of differences in detection rates across assay platforms, we conducted a tumor type-specific analysis for seven tumor types. In all tumor types, the prevalence of *HER2* amplification with F1CDx was markedly higher than that with the other four platforms.

Given that in the HERALD trial [6], patient enrollment was performed using G360CDx and the efficacy of T-DXd was demonstrated, the cutoff for *HER2* amplification in the companion diagnostic-approved G360CDx may serve as the gold standard in Japan. On the other hand, an ongoing expansion cohort of the HERALD trial is enrolling patients with *HER2* amplification confirmed by any pharmaceutical-approved CGP testing, and the results from this cohort are expected to provide further insight into tissue-selected populations (jRCT2080224635). The results of the HERALD expansion cohort might warrant close attention to assess the correlation between the detection of *HER2* amplification by F1CDx and the therapeutic efficacy of T-DXd.

In addition, results from the exploratory biomarker analyses of the DESTINY-PanTumor02 trial [22] showed that the overall response rate (ORR) in IHC 3+ cases and the ORR in *HER2* amplification-positive cases identified using the Guardant OMNI platform, which has analytical performance comparable to G360CDx, were similar (61.3% and 60.4%, respectively) [23]. On this basis, detection by F1CDx alone may carry the risk of designating patients as *HER2*-positive outside the fraction corresponding to IHC 3+, for whom treatment efficacy can be expected. To avoid this, additional IHC testing may be useful in cases with low copy numbers that are identified as *HER2* amplification-positive by CGP testing [24]. However, the range considered as low copy number is not sufficiently defined and may also vary depending on the panels. This range may require further validation, for example, through cross-validation or drug response assessment.

In this study, we also analyzed differences in co-occurring genetic alterations between the *HER2* amplification and *HER2* no-amplification groups. With respect to co-occurrence, gene mutations were more numerous and diverse in the *HER2* amplification group than in the *HER2* no-amplification group. Among these, a marked difference was observed in *KRAS* mutations, which were more frequent in the *HER2* no-amplification group (28%) than in the *HER2* amplification group (13%). In some tumor types, *KRAS* mutations and *ERBB2* (*HER2*) amplification have been reported to exhibit a nearly mutually exclusive relationship [25], which may have contributed to this bias. Clinically important, however, is the coexistence of *KRAS* mutations in the *HER2* amplification group, as multiple studies, including the tumor-agnostic MyPathway basket trial [22,26], have reported that *KRAS* mutations confer resistance to anti-*HER2* therapies. Although it is unclear if *KRAS* mutations are related to the efficacy of T-DXd from this study, this may be relevant in tumor types less responsive to T-DXd or in *HER2* amplification-positive cases with low copy numbers, where determination of the *KRAS* mutation status could be critical for treatment decisions. Further studies are required to confirm the correlation between *KRAS* mutations and efficacy of T-DXd, as well as to elucidate the detailed mechanisms involved.

Indeed, in the DESTINY-PanTumor02 and HERALD trials, almost no efficacy was observed in pancreatic cancer, where *KRAS* mutations are highly prevalent. Similarly, co-amplification of *HER2* and *CDK12* in breast cancer has been reported to be associated with poor prognosis and resistance to anti-*HER2* therapy (NGS enables assessment not only of the primary *HER2* gene amplification status but also of the coexistence of genomic alterations related to treatment resistance, and may provide novel therapeutic options, particularly in the presence of activating mutations) [20,27]. It also holds promise for advancing future diagnostic and therapeutic strategies.

Our analysis further suggests that CGP is often performed late in the treatment continuum, particularly in common cancers, where it is usually initiated after third-line therapy. This practice may limit timely access to targeted therapies and highlights the need to integrate CGP earlier in the diagnostic and treatment pathway [28,29]. Generally, treatment achievement ratio after EP evaluation is expected to be around 10% [30,31,32,33], which was lower than that reported in this study (*HER2* amplification group, 23.5%, and *HER2* no-amplification group, 17.8%) and could be attributed to the differences in the denominator.

The strengths of this study include the fact that this is a large study with approximately 90,000 cases, provides information on the prevalence of *HER2* amplification in a cross-section of Japanese patients from the C-CAT database, and provides details of the characteristics of the patients with *HER2* amplification. With the availability of limited population-based data on HER2 expression across different cancer types, the findings of this study contribute valuable information about HER2 positivity, especially with an increasing number of HER2 targeted therapies in the treatment landscape.

As a limitation, this is a retrospective study conducted using the C-CAT database and was restricted to patients who underwent CGP testing; the frequency of *HER2* amplification, clinical background, and mutational status cannot be readily generalized to the Japanese population. Another point of caution is that, in this study, *HER2* amplification was defined as the presence or absence of records on the C-CAT database and not up to copy number. Additionally, as evident from the detection rates of *HER2* amplification across assay platforms, the cutoff settings for gene amplification were not uniform (F1CDx [≥6 copies], NOP [≥8 copies], GMTOP [≥6 copies]). In particular, F1CDx is set to capture *HER2* amplification positivity more broadly. Given that approximately 70% of the nearly 90,000 cases in this study were tested with F1CDx, it should be noted that this has a certain influence on the estimated prevalence. In addition, there may be an option to not enter data into the C-CAT database, which may have resulted in missing data. However, these missing entries occur randomly in patients with *HER2* amplification and in those with *HER2* no-amplification; therefore, the effects of biases may be small. The specific copy number of *HER2* amplification could not be determined because it was not recorded in this database. Therefore, according to the copy number for each CGP platform, prospective validation of the usefulness of approved anti-*HER2* drugs (particularly T-DXd) in patients with *HER2* amplification is needed. Further, it is assumed that fresh pathological specimens were used for IHC and FISH testing, whereas CGP testing may have been performed on specimens obtained at different time points. These specimens may include long-term stored specimens, specimens re-biopsied after chemotherapy, or blood-based CGP samples. HER2 gene and protein expression may vary under these conditions. Lastly, it was not possible to assess institutional differences in test or platform selection within this population.

## 5. Conclusions

In conclusion, in this nationwide study of 89,374 Japanese patients with solid tumors, *HER2* amplification was identified in 5.7%, with the highest prevalence in esophagus/stomach and urothelial cancers. Detection rates differed markedly by assay modality (tissue CGP 6.5% vs. plasma CGP 1.8%) and across assay platforms. Notably, F1CDx, which accounted for most tests, showed a substantially higher detection rate (7.4%) than the other four platforms (1.5–2.3%); accordingly, interpretation of the observed prevalence may need to take this distribution into account. As a clinical consideration, when using CGP-based *HER2* amplification results to guide patient selection for anti-*HER2* therapy, cautious interpretation by an EP may be warranted.

## Figures and Tables

**Table 1:** Patient characteristics by the *HER2* amplification status.

	Item	ALL (*N* = 89,374)	*HER2* Amp (*N* = 5119)	*HER2* No-Amp (*N* = 84,255)
**Basic information**	**Age (years), mean [SD]**	62.5 [12.5]	62.5 [12.0]	62.5 [12.5]
	**Sex**			
	Male	44,611 (49.9%)	2368 (46.3%)	42,243 (50.1%)
	Female	44,759 (50.1%)	2751 (53.7%)	42,008 (49.9%)
	NA	4 (0.0%)	0 (0.0%)	4 (0.0%)
	**Smoking**			
	Yes	39,483 (44.2%)	2275 (44.4%)	37,208 (44.2%)
	No	45,060 (50.4%)	2608 (50.9%)	42,452 (50.4%)
	NA	4831 (5.4%)	236 (4.6%)	4595 (5.5%)
	**Metastasis**			
	Yes	79,962 (89.5%)	4789 (93.6%)	75,173 (89.2%)
	No	7366 (8.2%)	224 (4.4%)	7142 (8.5%)
	NA	2046 (2.3%)	106 (2.1%)	1940 (2.3%)
	**Metastasis area**			
	Bone	15,838 (17.7%)	840 (16.4%)	14,998 (17.8%)
	Peritoneum	17,595 (19.7%)	897 (17.5%)	16,698 (19.8%)
	Liver	29,410 (32.9%)	1982 (38.7%)	27,428 (32.6%)
	Lung	25,792 (28.9%)	1688 (33.0%)	24,104 (28.6%)
	Lymph nodes/lymph vessels	31,774 (35.6%)	2273 (44.4%)	29,501 (35.0%)
**CGP Testing**	**ALL**			
	**Tissue ALL**	73,960 (82.8%)	4839 (94.5%)	69,121 (82.0%)
	NCC OncoPanel	8584 (9.6%)	181 (3.5%)	8403 (10.0%)
	FoundationOne CDx	62,299 (69.7%)	4586 (89.6%)	57,713 (68.5%)
	GenMineTOP	3077 (3.4%)	72 (1.4%)	3005 (3.6%)
	**Liquid ALL**	15,414 (17.2%)	280 (5.5%)	15,134 (18.0%)
	FoundationOne Liquid CDx	13,719 (15.4%)	254 (5.0%)	13,465 (16.0%)
	Guardant360 CDx	1695 (1.9%)	26 (0.5%)	1669 (2.0%)
**Sampling**	**Sample collection method** **(denominator is tissue ALL)**			
	Surgery	45,137 (61.0%)	2868 (59.3%)	42,269 (61.2%)
	Biopsy	28,566 (38.6%)	1960 (40.5%)	26,606 (38.5%)
	Other	245 (0.3%)	10 (0.2%)	235 (0.3%)
	Unknown	12 (0.0%)	1 (0.0%)	11 (0.0%)
	**Specimen collection site** **(denominator is tissue ALL)**			
	Primary lesion	50,458 (68.2%)	3291 (68.0%)	47,167 (68.2%)
	Metastatic lesion	23,169 (31.3%)	1531 (31.6%)	21,638 (31.3%)
	Unknown	333 (0.5%)	17 (0.4%)	316 (0.5%)
**Treatment**	**Treatment line (Pre-EP)**			
	1L	20,260 (22.7%)	1031 (20.1%)	19,229 (22.8%)
	2L	22,048 (24.7%)	1214 (23.7%)	20,834 (24.7%)
	3L	16,720 (18.7%)	928 (18.1%)	15,792 (18.7%)
	4L	10,438 (11.7%)	648 (12.7%)	9790 (11.6%)
	≥5L	13,649 (15.3%)	1015 (19.8%)	12,634 (15.0%)
	NA	6259 (7.0%)	283 (5.5%)	5976 (7.1%)
	**Prior *HER*2-targeted therapy**	2206 (2.5%)	881 (17.2%)	1325 (1.6%)
	Trastuzumab	1504 (1.7%)	805 (15.7%)	699 (0.8%)
	Trastuzumab emtansine	568 (0.6%)	276 (5.4%)	292 (0.3%)
	T-DXd	1276 (1.4%)	388 (7.6%)	888 (1.1%)
	Pertuzumab	735 (0.8%)	359 (7.0%)	376 (0.4%)
	Lapatinib	214 (0.2%)	115 (2.2%)	99 (0.1%)

Data are shown as *n* (%) unless stated otherwise. The bold texts are used for subheadings. 1L: first-line; 2L: second-line; 3L: third-line; 4L: fourth-line; 5L: fifth-line; Amp: amplification; CGP: comprehensive genomic profiling; EP: expert panel; *HER2*: human epidermal growth factor receptor 2; NA: not applicable; SD: standard deviation; T-DXd: trastuzumab deruxtecan.

**Table 2:** Evaluation of expert panel-recommended genomically matched drug treatments for the *HER2* amplification group and the *HER2* no-amplification group.

Tumor Type	*HER2* Status	Total (*N*)	Presented New Therapeutic Drug Options			Administered the Presented Therapeutic Agent *		
			Yes	No	Unknown	Yes	No	Unknown
**ALL**	No-amp	84,255	32,798 (38.9%)	43,442 (51.6%)	8015 (9.5%)	5840 (17.8%)	22,597 (68.9%)	4361 (13.3%)
	Amp	5119	3183 (62.2%)	1421 (27.8%)	515 (10.1%)	748 (23.5%)	1997 (62.7%)	438 (13.8%)
**Ampulla of Vater**	No-amp	477	206 (43.2%)	231 (48.4%)	40 (8.4%)	14 (6.8%)	156 (75.7%)	36 (17.5%)
	Amp	54	25 (46.3%)	25 (46.3%)	4 (7.4%)	6 (24.0%)	15 (60.0%)	4 (16.0%)
**Biliary Tract**	No-amp	7293	2741 (37.6%)	3905 (53.5%)	647 (8.9%)	435 (15.9%)	1913 (69.8%)	393 (14.3%)
	Amp	673	425 (63.2%)	179 (26.6%)	69 (10.3%)	79 (18.6%)	292 (68.7%)	54 (12.7%)
**Bladder/** **Urinary Tract**	No-amp	1327	598 (45.1%)	609 (45.9%)	120 (9.0%)	109 (18.2%)	418 (69.9%)	71 (11.9%)
	Amp	157	98 (62.4%)	40 (25.5%)	19 (12.1%)	19 (19.4%)	66 (67.3%)	13 (13.3%)
**Bowel**	No-amp	14,085	6433 (45.7%)	6480 (46.0%)	1172 (8.3%)	811 (12.6%)	4641 (72.1%)	981 (15.2%)
	Amp	803	526 (65.5%)	195 (24.3%)	82 (10.2%)	174 (33.1%)	257 (48.9%)	95 (18.1%)
**Breast**	No-amp	6081	2658 (43.7%)	2627 (43.2%)	796 (13.1%)	648 (24.4%)	1505 (56.6%)	505 (19.0%)
	Amp	639	382 (59.8%)	178 (27.9%)	79 (12.4%)	111 (29.1%)	203 (53.1%)	68 (17.8%)
**Cervix**	No-amp	2036	704 (34.6%)	1172 (57.6%)	160 (7.9%)	165 (23.4%)	465 (66.1%)	74 (10.5%)
	Amp	141	95 (67.4%)	38 (27.0%)	8 (5.7%)	19 (20.0%)	64 (67.4%)	12 (12.6%)
**CNS/Brain**	No-amp	2126	702 (33.0%)	1198 (56.3%)	226 (10.6%)	143 (20.4%)	463 (66.0%)	96 (13.7%)
	Amp	32	19 (59.4%)	10 (31.3%)	3 (9.4%)	3 (15.8%)	15 (78.9%)	1 (5.3%)
**Esophagus/** **Stomach**	No-amp	4790	1595 (33.3%)	2720 (56.8%)	475 (9.9%)	187 (11.7%)	1200 (75.2%)	208 (13.0%)
	Amp	709	418 (59.0%)	215 (30.3%)	76 (10.7%)	93 (22.2%)	268 (64.1%)	57 (13.6%)
**Head and Neck**	No-amp	2680	806 (30.1%)	1641 (61.2%)	233 (8.7%)	130 (16.1%)	608 (75.4%)	68 (8.4%)
	Amp	231	144 (62.3%)	72 (31.2%)	15 (6.5%)	35 (24.3%)	99 (68.8%)	10 (6.9%)
**Kidney**	No-amp	679	214 (31.5%)	397 (58.5%)	68 (10.0%)	40 (18.7%)	156 (72.9%)	18 (8.4%)
	Amp	17	8 (47.1%)	7 (41.2%)	2 (11.8%)	0 (0.0%)	8 (100.0%)	0 (0.0%)
**Liver**	No-amp	755	195 (25.8%)	475 (62.9%)	85 (11.3%)	42 (21.5%)	134 (68.7%)	19 (9.7%)
	Amp	30	19 (63.3%)	7 (23.3%)	4 (13.3%)	4 (21.1%)	14 (73.7%)	1 (5.3%)
**Lung**	No-amp	5069	2330 (46.0%)	2158 (42.6%)	581 (11.5%)	706 (30.3%)	1336 (57.3%)	288 (12.4%)
	Amp	348	237 (68.1%)	75 (21.6%)	36 (10.3%)	58 (24.5%)	146 (61.6%)	33 (13.9%)
**Ovary/Fallopian Tube**	No-amp	4562	1753 (38.4%)	2398 (52.6%)	411 (9.0%)	327 (18.7%)	1173 (66.9%)	253 (14.4%)
	Amp	379	240 (63.3%)	108 (28.5%)	31 (8.2%)	34 (14.2%)	177 (73.8%)	29 (12.1%)
**Pancreas**	No-amp	13,730	5311 (38.7%)	6994 (50.9%)	1425 (10.4%)	408 (7.7%)	4242 (79.9%)	661 (12.4%)
	Amp	246	152 (61.8%)	66 (26.8%)	28 (11.4%)	22 (14.5%)	113 (74.3%)	17 (11.2%)
**Peritoneum**	No-amp	577	186 (32.2%)	355 (61.5%)	36 (6.2%)	39 (21.0%)	126 (67.7%)	21 (11.3%)
	Amp	13	8 (61.5%)	4 (30.8%)	1 (7.7%)	0 (0.0%)	7 (87.5%)	1 (12.5%)
**Prostate**	No-amp	5421	1840 (33.9%)	3167 (58.4%)	414 (7.6%)	649 (35.3%)	966 (52.5%)	225 (12.2%)
	Amp	89	45 (50.6%)	38 (42.7%)	6 (6.7%)	12 (26.7%)	29 (64.4%)	4 (8.9%)
**Skin**	No-amp	1290	554 (42.9%)	641 (49.7%)	95 (7.4%)	143 (25.8%)	367 (66.2%)	44 (7.9%)
	Amp	91	63 (69.2%)	21 (23.1%)	7 (7.7%)	19 (30.2%)	38 (60.3%)	6 (9.5%)
**Soft Tissue**	No-amp	3256	1012 (31.1%)	1943 (59.7%)	301 (9.2%)	188 (18.6%)	726 (71.7%)	98 (9.7%)
	Amp	37	19 (51.4%)	13 (35.1%)	5 (13.5%)	5 (26.3%)	11 (57.9%)	3 (15.8%)
**Thymus**	No-amp	624	149 (23.9%)	430 (68.9%)	45 (7.2%)	25 (16.8%)	109 (73.2%)	15 (10.1%)
	Amp	3	13 (38.2%)	18 (52.9%)	3 (8.8%)	2 (15.4%)	9 (69.2%)	2 (15.4%)
**Thyroid**	No-amp	883	515 (58.3%)	274 (31.0%)	94 (10.6%)	161 (31.3%)	308 (59.8%)	46 (8.9%)
	Amp	9	6 (66.7%)	1 (11.1%)	2 (22.2%)	3 (50.0%)	2 (33.3%)	1 (16.7%)
**Uterus**	No-amp	2810	1031 (36.7%)	1517 (54.0%)	262 (9.3%)	160 (15.5%)	751 (72.8%)	120 (11.6%)
	Amp	258	169 (65.5%)	66 (25.6%)	23 (8.9%)	28 (16.6%)	124 (73.4%)	17 (10.1%)
**Other**	No-amp	2192	823 (37.5%)	1163 (53.1%)	206 (9.4%)	212 (25.8%)	528 (64.2%)	83 (10.1%)
	Amp	102	62 (60.8%)	29 (28.4%)	11 (10.8%)	19 (30.6%)	35 (56.5%)	8 (12.9%)

Data are shown as *n* (%) unless stated otherwise. * “Administered the presented therapeutic agent” = “Yes.”; amp, amplification; CNS: central nervous system; *HER2*: human epidermal growth factor receptor 2; no-amp, no-amplification.

## Data Availability

The raw data supporting the conclusions of this article will be made available by the corresponding author and the funder, Daiichi Sankyo Co., Ltd., on request.

## Supplementary Materials

The following supporting information can be downloaded at: https://www.mdpi.com/article/10.3390/curroncol33040195/s1, Figure S1: Heat map of copy number alteration A) with and B) without *HER2* amplification; Table S1: Patient characteristics by tumor type; Table S2: Prevalence of *HER2* amplification according to the OncoTree hierarchical classification system; Table S3: Percentage of *HER2* amplification detected by CGP according to sample type; Table S4: Comparison of the *HER2* status between prior companion diagnostic testing and CGP testing in selected tumor types; Table S5: Relationship between the *HER2* status from CGP testing and predictive biomarker statuses from prior companion diagnostic testing in selected tumor types; Table S6: Detected driver gene fusions/rearrangements in cases with and without *HER2* amplification; Table S7: Evaluation of expert panel-recommended genomically matched drug treatments (treatment policy and applied drug groups) for the *HER2* amplification group and the *HER2* no-amplification group.
