# Supplementary figures and images for: Tumor-Agnostic Landscape with HER2 Amplification in Japan: Real-World Prevalence and Implications for Targeting HER2
Source: Curr Oncol. 2026 Mar 30;33(4):195. doi: 10.3390/curroncol33040195 (PMC13115398; doi:10.3390/curroncol33040195)

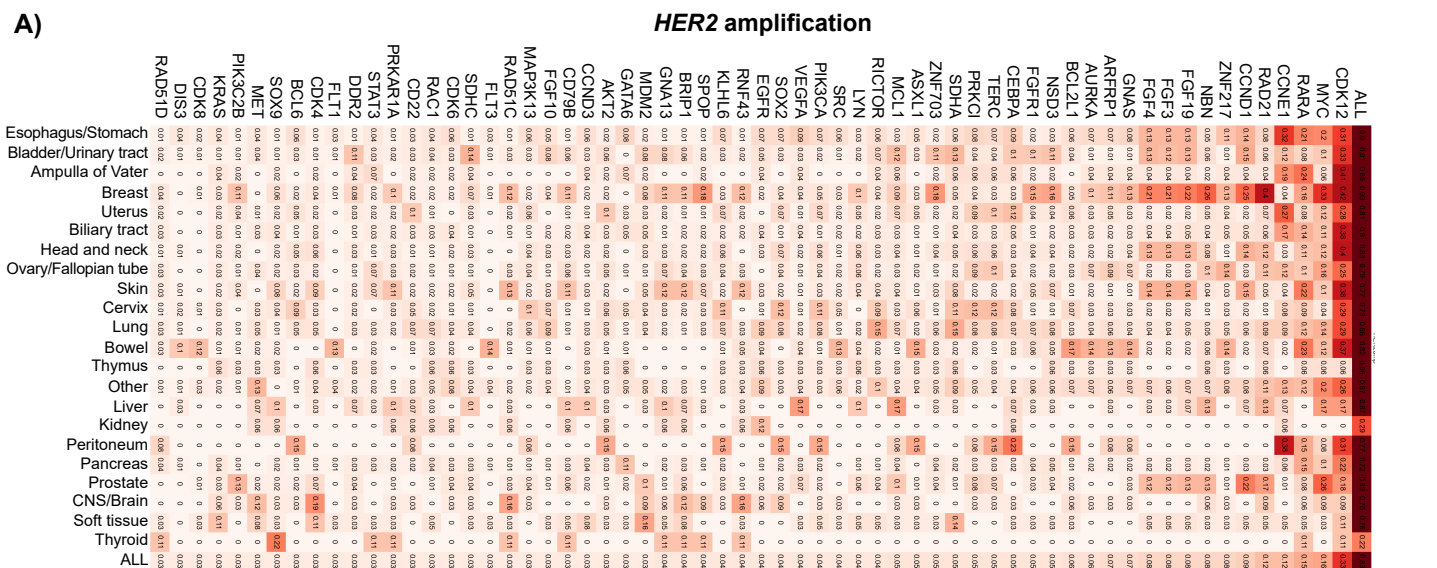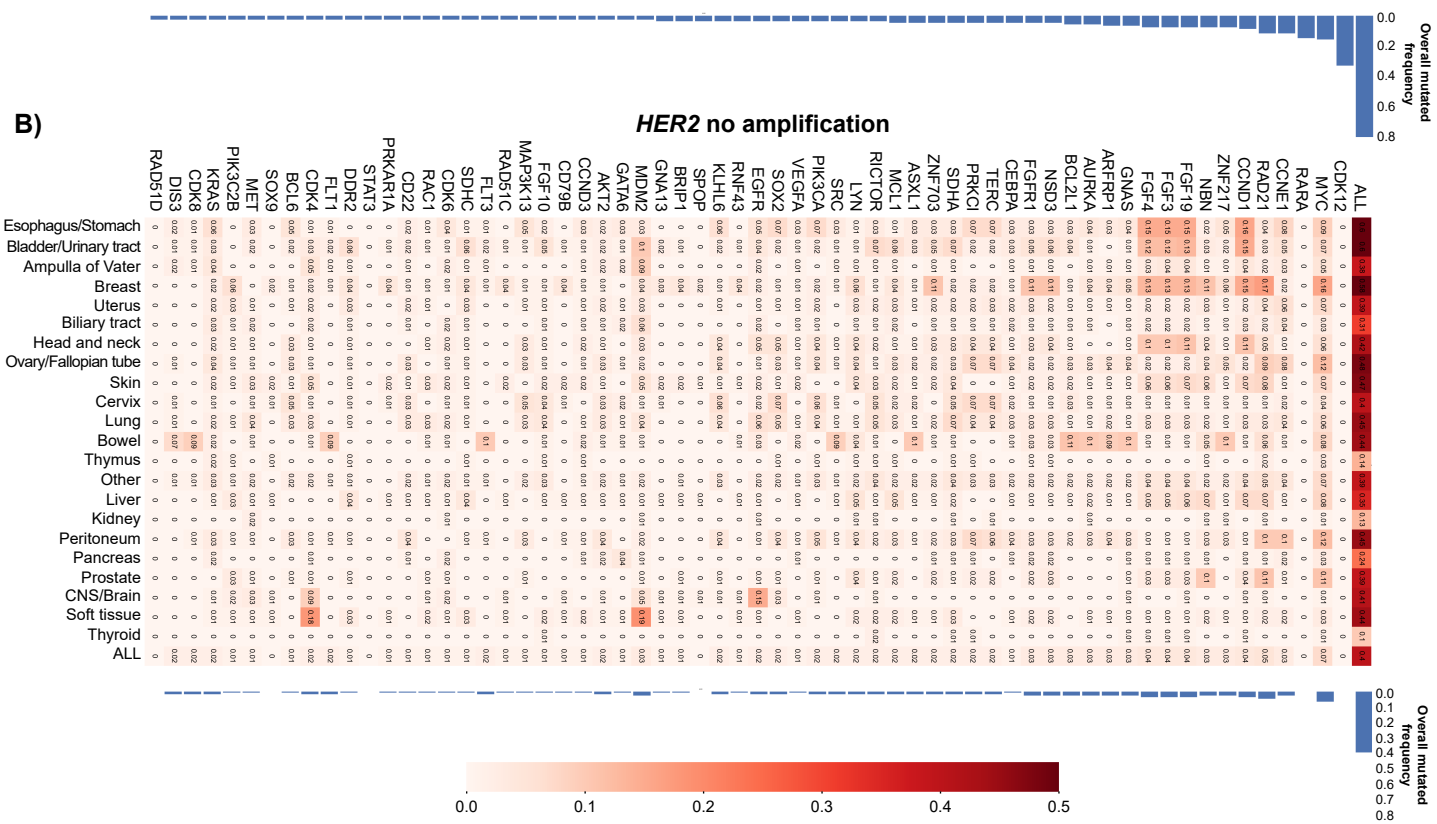

Supplement: Supplementary file 1 [file curroncol-33-00195-s001.zip › curroncol-4180604-Figure S1.pdf]
